# House Sparrow (*Passer domesticus*) escape behavior is triggered faster in smaller settlements

**DOI:** 10.1038/s41598-022-26988-0

**Published:** 2023-02-13

**Authors:** Michelle García-Arroyo, Ian MacGregor-Fors, Javier Quesada, Antoni Borràs, Laia Colomé-Menoyo, Juan Carlos Senar

**Affiliations:** 1 Ecosystems and Environment Research Programme, Faculty of Biological and Environmental Sciences, University of Helsinki, Niemenkatu 73, 15140 Lahti, Finland; 2 Museu de Ciències Naturals de Barcelona, Barcelona, Catalonia Spain

**Keywords:** Behavioural ecology, Urban ecology, Ecology, Evolution, Zoology

## Abstract

A recurrent behavioral trait model to study adaptation to urban environments is the flight initiation distance (FID), measured as the distance at which animals flee from an approaching threat. It has previously been shown that urban birds display shorter FID than their non-urban (rural) counterparts. However, discerning whether this is the result of habituation to human presence and frequentation, or of ecological factors related to the size of the city (considered as “systemic habituation”), has not yet been addressed. In this study, we analyzed House Sparrow (*Passer domesticus*) FIDs in a network of 26 small towns and villages within the same region in northeastern Spain. Our aim was to relate FID to human population density and settlement size. If the habituation to human presence hypothesis was supported, we should expect FIDs to decrease with the density of the human population across the human settlements, since this type of habituation is related to the rate of human exposure and this is proportional to human density. However, if the systemic habituation hypothesis was supported, FIDs should instead relate to the size of the human settlements, as the abundance of predators, similarly to other ecological variables, is often proportional to the size of towns. Results showed House Sparrows to be bolder in larger human settlements, but not necessarily the ones with a higher density of human population. This supports the idea that the fact that urban birds display shorter FIDs than their rural counterparts is the result of systemic ecological factors rather than the results of a simple habituation to humans.

## Introduction

Behavioral plasticity is an important wildlife trait, occasionally triggering individuals to be able to endure and even thrive under novel conditions, such as the wide array of anthropogenic environmental modifications^1^. Many behavioral changes and traits have been linked with species that currently thrive in urban settings, such as shifts in diet, increased boldness, and reduced neophobia^2,3^. Among the broadly studied approaches to understanding such types of plasticities in behavioral ecology a recurrent trait model is the flight initiation distance (FID) or flushing behavior^4^. FID is described as the distance at which animals flee from an approaching potential threat^5,6^. This trait has shown to be a particularly good model to study adaptation to new environments, given that evidence suggests it to be adaptive, as individuals adjust the perception of the predation risk with the intensity of the threat^7^. Such “anti-predatory”/escape behavior has been utilized to determine adequate buffer distances in areas of human–wildlife conflicts^8–10^, but has also been used to assess differences in behavior among populations between diverse predator characteristics, environmental conditions, or disturbance intensities^11–13^.

A key feature in understanding species plasticity and related adaptations, is assessing the population’s variation of FIDs^14^. House Sparrow FIDs have been found to be shorter in urban environments when compared with those of individuals from non-urban areas in European studies assessing several bird species^15–17^. Studies have also reported that individuals display bolder behaviors in larger urban areas^18–20^. However, it is still unclear which are the mechanisms behind such a pattern. A general widely accepted hypothesis is that this variation may be the result of habituation of the species to human presence, considering habituation as the learned behavior where individuals’ responses to specific stimuli decrease over time due to constant exposure to the stimuli^21–23^. This hypothesis is supported by data showing that individuals alter their FID based on previous experiences and according to the rate of human exposure^23,24^. The fact that FIDs can be negatively correlated to time since colonization in a city has also been used to support this hypothesis^25,26^.

Alternatively, shorter avian FIDs in urban settlements compared to non-urban (rural) ones could also be a consequence of ecological factors related, for example, to food availability and predictability, competition, and a reduced presence of predators in urban habitats^27,28^. For instance, predation is relaxed in large urban settlements and may explain the increased boldness of prey species in urbanized areas^29^, also having an impact on FIDs^30^. Hence, the size of the city per se may play an important role in explaining the reduction in FIDs given the landscape heterogeneity nature of increasingly growing urban areas, particularly if the home range of one species is completely covered by the urban area or includes surrounding systems^31^.

Discerning between the relative importance of habituation to human presence and frequentation, and ecological factors related to the size of the city (considered as “systemic habituation” in this study), has not yet been addressed. The relative importance of these two alternative hypotheses (i.e., habituation to human presence, systemic habituation) could be tested by relating FIDs across urban settlements of different sizes within the same region. If the habituation to human presence hypothesis is supported, we should expect FIDs to decrease with the density of the human population across the human settlements, since this type of habituation is related to the rate of human exposure and this is proportional to human density^24^. However, if the systemic habituation hypothesis is supported, FIDs should instead relate to the size of the human settlements, as the abundance of predators, similarly to food availability or disease prevalence, is often proportional to the size of towns^27,28,32^. To our knowledge, this is the first study to address both hypotheses in a comparative approach across cities.

In this study, we assessed the relative effect of settlement size and human density on FIDs of House Sparrows *Passer domesticus* across an array of 26 different-sized urban settlements within the same region in the Catalan Central Depression (northeastern Iberian Peninsula). We focused on the House Sparrow since it has been shown to be an ideal model species to study FIDs^18,19,33,34^. Previous studies have reported that individuals display a bolder behavior the larger the urban area^20,35–37^, and House Sparrow FIDs have also been found to be shorter in urban environments when compared with those of individuals from non-urban areas^15–17^. We analyzed FID variability in the same region to avoid collateral effects on FID related to latitudinal, climatic or cultural effects^38^. We focused on the Catalan Central Depression because the species extends across the area of all settlements^39^. Yet, smaller settlements are embedded by an agricultural-forested matrix, where sparrows inhabit several land uses in the landscape that varies as settlement size increases^40^.

## Methods

**Figure 1:**
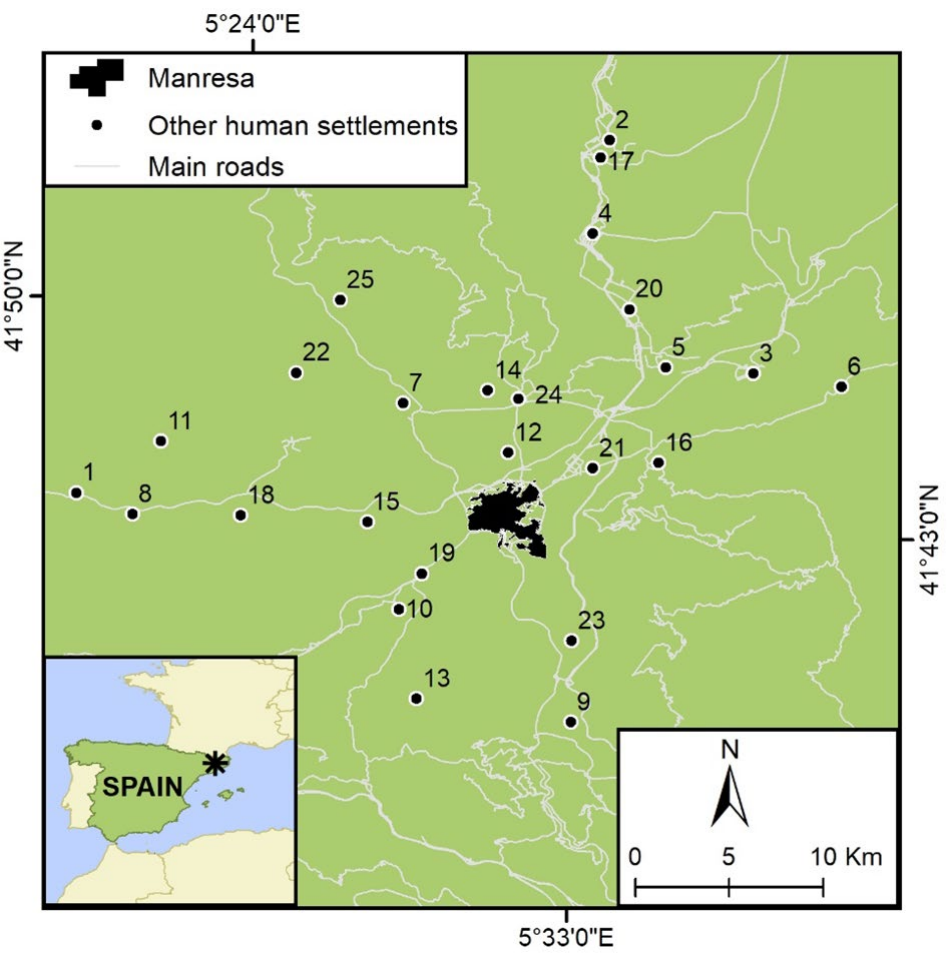
Geographic location of the studied human settlements. The numbers correspond to those indicated in Table 1. This figure was drawn using ArcGIS [GIS software] Version 10.8.0.12790 (http://www.esri.com).

This study was performed in the county (*comarca*) of El Bages (1092 km^2^; 180,500 inhabitants^41^), located in the Catalan Central Depression, Catalonia, northeastern Spain (Fig. 1). It is composed of 30 municipalities and its capital is the city of Manresa. Overall, the urban areas of this region have been established and influenced by the presence of the rivers Llobregat and Cardener and the hilly topography that surrounds it^42,43^. Human settlements in the study area vary in size and population density (Table 1).

**Table 1:** Size, total human population density, distance to Manresa, and House Sparrow abundance of the studied human settlements. Population data was obtained from the Statistical Institute of Catalonia^41^.

	Size (km^2^)	Human pop. density	Dist. to Manresa (km)	House Sparrow abundance	Settlement in Fig. 1
Manresa	6.64	11,445.78	0.00	0.53	–
Sant Vicenç de Castellet	2.28	3682.02	6.77	0.41	23
Santpedor	2.10	3176.19	6.11	0.49	24
Sant Fruitós de Bages	1.72	3301.74	4.67	0.52	21
Navarcles	1.34	4250.00	6.73	0.48	16
Súria	1.22	5082.79	13.15	0.27	25
Artés	1.13	4851.33	12.17	0.49	3
Sallent	1.13	5561.95	11.54	0.36	20
El Calvet	0.69	1926.09	6.62	0.41	10
Navàs	0.99	5900.00	19.48	0.51	17
Pineda de Bages	0.52	1784.62	3.38	0.57	12
Balsareny	0.48	6418.75	15.40	0.44	4
Castellbell i el Vilar	0.45	8246.67	10.95	0.33	9
Callús	0.34	5138.24	7.26	0.42	7
Cabrianes	0.33	1048.48	9.56	0.55	5
Salelles	0.29	937.93	4.54	0.40	19
Mirador de Monserrat	0.28	1667.86	6.57	0.49	14
Calders	0.15	5813.33	15.00	0.34	6
Sant Mateu de Bages	0.14	678.57	11.20	0.22	22
Fonollosa	0.12	2091.67	14.19	0.44	11
Aguilar de Segarra	0.10	1370.00	17.12	0.31	1
l'Ametlla de Merola	0.10	2370.00	20.50	0.44	2
Marganell	0.03	3800.00	10.39	0.22	13
Rajadell	0.03	9566.67	10.37	0.13	18
Castellar	0.01	8000.00	14.83	0.14	8
Monistrolet	0.01	4700.00	5.49	0.42	15

The same observer (LC-M) recorded House Sparrow FIDs across 26 human settlements, including the capital of the region, Manresa (Table 1; Fig. 1) from August to December 2012. Field surveys were performed on days with no rain and only mild perceived wind, always focusing on individuals that were on ground-level, from 08:00 to 18:00 h. We recorded 3–5 FID measurements per settlement (average 3.46 ± 0.57 SD). Following standard FID procedures^4,6^, LC-M approached individuals only if she could walk in a straight line without obstacles towards the focal bird. Once the bird fled, she measured the distance between her and the location where the House Sparrow took flight with a wooden surveyor’s wheel. Notably, for this study, we selected data from independent flocks that were recorded at different times (at least 10 min difference between data collection) to minimize pseudo-replication issues. We explored the correlation between the FIDs and the time of the day and date in which they were recorded, but since we only found weak correlations (FID, date: r = 0.098, *P* = 0.0227; FID, time of day: r = − 0.132, *P* = 0.104), we did not include these variables in further analyses.

In addition to human population density and settlement size (both independent variables behind our main hypotheses), we considered two additional independent covariates given their potential in explaining House Sparrow FIDs: distance to Manresa and House Sparrow abundance. We considered the distance to Manresa (the largest city in the region) as we assume that given its size, it may hold the largest House Sparrow population in our study area. Previous studies have shown that House Sparrows have broader home ranges and disperse easily across agricultural landscapes^44^ which is the type of matrix the studied human settlements are embedded in. In relation to House Sparrow abundances, we included it due to the evidence of density-dependent responses in their urban populations. We obtained relative abundance data for House Sparrows in our study area from the Catalan Winter Bird Atlas^45^. Abundance data was modelled through maximum-entropy modelling approaches using MAXENT considering a 1 km^2^ resolution, with values ranging from 0 to 1. Raw abundance data was recorded through standardized monitoring programs based on 3 km line transects^45^. To determine the distance between the studied settlements and Manresa, we measured the extent of each town by delimiting the polygon of their built continuum (from which we retrieved their size), following the parameters of building clustering and communication specified by MacGregor-Fors^46^ and Lemoine-Rodríguez et al.^47^. Briefly, this procedure consists of drawing the boundaries of the urbanized continuum by visually identifying the continuity of their built infrastructure, taking into account the connectivity (through identifiable paved roads) of built aggregations, using high-resolution satellite imagery. Once these polygons were drawn, we measured the distance between the centroid of the polygon of Manresa to the centroid of the rest of the studied settlements (Table 1). We retrieved human population data from the Statistical Institute of Catalonia^41^. Human density was calculated by dividing the reported human population for each settlement and its size. We recognize that the reported population may extend the limits of the delineated polygons; yet, most of such populations are scattered across their peri- and extra-urban areas, not representing the core of their populations.

In order to test the relationship between human settlement size, human population density, House Sparrow abundance, and distance to Manresa (independent variables) with House Sparrow FIDs, we performed a set of 17 linear models (LM in R^48^) taking into account all possible combinations among the four independent variables while considering potential interaction between human settlement size and population density. Due to the nature of the variables and their distribution, we log-transformed House Sparrow FIDs, human population density, and settlement size. We assured that independent variables were not correlated to avoid potential multicollinearity issues (all correlation coefficients r <|0.48|). Model selection was based on the Akaike’s Information Criterion (AICc, corrected for small sample sizes), using the relative differences of criterion results in relation to the smaller value (ΔAICc) following Burnham and Anderson^49^. We considered models differing by 2 ΔAICc from the top model as those that were most supported by our data^49^, followed by the proportion of predictive power that can be found in the model (AICcWt).

## Results

**Figure 2:**
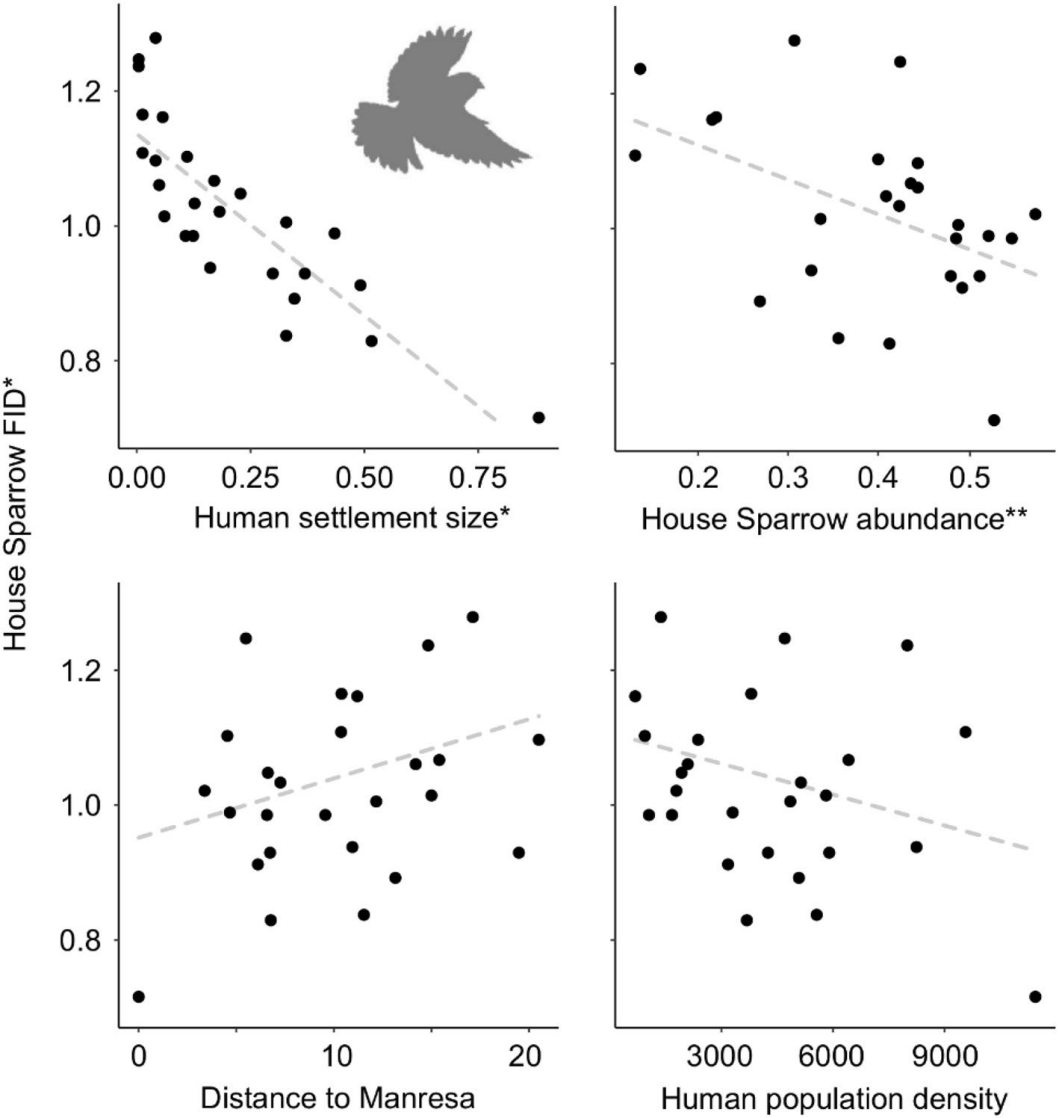
Relationship between human population density, settlement size, distance to Manresa, and House Sparrow abundance with sparrow FIDs. Segmented lines represent data tendencies in each plot. *Variables that were analyzed and are displayed as log_10_ transformed. **House Sparrow abundance data corresponds to modeled relative abundances (see “Methods” section for further details).

Average FID per settlement was 10.0 m (± 0.6 SE), ranging from 18.0 m in Aguilar de Segarra to 4.2 m in downtown Manresa (Barri Vell). The model solely considering human settlement size was the fittest one, showing considerable proportion of predictive power (AICcWt = 0.44), with the next most parsimonious model having a ΔAIC value > 2 (F_1,24_ = 56.6, *P* < 0.001; r^2^ = 0.70) (Fig. 2). Interestingly, the models considering human settlement size and the other three predictor variables, independently, were the best fit models, although all > 2 ΔAIC value: House Sparrow abundance (ΔAIC = 2.19), distance to Manresa (ΔAIC = 2.40), and human density (ΔAIC = 2.41) (Table 2).

**Table 2:** Models relating human population density (hum.dens), settlement size (sett.size), distance to Manresa (dist.Manresa), and House Sparrow abundance (HSabund.) with sparrow FIDs (HS.FID) in 26 settlements of the region of Manresa, Spain.

Model names	Parameters	AICc	ΔAICc	AICcWt
HS.FID ~ sett.size	3	− 56.15	0.00	0.44
HS.FID ~ sett.size + HSabund	4	− 53.96	2.19	0.15
HS.FID ~ sett.size + dist.Manresa	4	− 53.75	2.40	0.13
HS.FID ~ sett.size + hum.dens	4	− 53.74	2.41	0.13
HS.FID ~ sett.size + hum.dens. + HSabund	5	− 51.97	4.18	0.05
HS.FID ~ sett.size*hum.dens	5	− 51.07	5.09	0.03
HS.FID ~ sett.size + hum.dens. + dist.Manresa	5	− 50.89	5.26	0.03
HS.FID ~ sett.size + hum.dens. + dist.Manresa + HSabund	6	− 48.89	7.27	0.01
HS.FID ~ sett.size*hum.dens. + HSabund	6	− 48.85	7.30	0.01
HS.FID ~ sett.size*hum.dens. + dist-Manresa	6	− 47.94	8.21	0.01
HS.FID ~ sett.size*hum.dens. + dist.Manresa + HSabund	7	− 45.43	10.72	0.00
HS.FID ~ hum.dens. + HSabund	4	− 35.22	20.93	0.00
HS.FID ~ HSabund	3	− 31.22	24.93	0.00
HS.FID ~ HSabund. + dist.Manresa	4	− 29.57	26.59	0.00
HS.FID ~ hum-dens. + dist.Manresa	4	− 28.94	27.21	0.00
HS.FID ~ dist.Manresa	3	− 27.76	28.39	0.00
HS.FID ~ hum-dens	3	− 27.72	28.44	0.00

## Discussion

The tolerance of birds to human disturbances is critical for their survival in cities, which is why some behavioral responses have been found to vary between populations of the same species living in different environments^50,51^. Results from this study provide evidence that House Sparrows are bolder in larger human settlements, with a less important role of variables like House Sparrow abundance, distance to main urban settlement (Manresa), and human population density, independently. This result stresses that the shorter House Sparrow FIDs in larger urban settings is mostly related to the ecological factors related to the size of the city (i.e. systemic habituation hypothesis), while when contrasted to settlement size, the recorded trends for House Sparrow abundance (negative), distance to Manresa (positive), and human population density (negative) seem rather weak or less informative.

Having found such a clear relationship between human settlement size and House Sparrow FIDs in this study was somehow surprising, as many studies in the past have explained many of the behaviors of the species through habituation to human presence^21–24^. Given the particular way in which we measured the size of the urban areas (i.e., enclosing the urban continuum instead of adhering to administrative divisions), it allows us to capture with more detail the ecological reality of what birds might experience in terms of ecotones. This approximation could be behind the unusual importance in the correlation we found, when compared with more traditional urban variables related to bird behavior^1^.

Evidently, the size of human settlements could imply a plethora of potential drivers that together explained the recorded result. The most evident to us is the decrease of potential predators in urban settings^28,29^, which could relax their general vigilance behavior and hence reduce FID^27^. Cats could potentially be an urban predator increasing predation risk in cities, but this is not the case in cities and villages in NE Spain, where cats are not so popular as in other regions of Europe. For instance, total number of cats in 2014 in Manresa was only 218, which means a density of 0.33 cats per ha (https://www.regio7.cat/manresa/2019/02/08/els-animals-censats-creixen-84-50118018.html, accessed 11/11/2022). Additionally, in small towns in NE Spain, most of the cats stay at home.

In addition to a reduction in predation risk in cities, it is known that competition and even parasitism can be relaxed in urban areas when contrasted with non-urban agricultural settings, for instance^32,52^. Finally, there are also potential spatial factors that could be embedded in our settlement size variable, such as: (1) the environmental heterogeneity that occurs in increasingly larger settings^40^, (2) differences in urban and non-urban territory sizes, and (3) the fact that small settlements may include non-urban land-uses within the territory of individuals, mixing the potential effects of land-uses in their behavior. All of these factors, as well as others we may be overseeing, could indirectly affect the pace of life of urban House Sparrows and influence their FIDs; yet, future empirical evidence is needed to test this.

Although the models considering House Sparrow abundance, distance to Manresa, and human population density only showed a weak statistical signal in the light of the strong relationship with settlement size alone, these variables should not be overlooked in the future, even more so in single-city/town studies. All of these variables have been shown in the past to relate in one way or another to the behavior and ecology of the House Sparrow^20,33,53^, leading us to consider that they may be influencing it on a local scale.

Altogether, our results show that the larger the settlements, the shorter the FIDs of House Sparrows, suggesting that the difference found in previous studies between urban and non-urban (rural) localities could be the result of a systemic habituation process. As previously shown by Vincze et al.^19^ although individuals from both urban and non-urban populations may respond in a similar way to human disturbance, urban individuals habituate faster, thus resulting in behavioral differences that in turn can be reflected in varying conducts, such as perceived risk and flight initiation decisions. Exactly how urbanization and differing urban conditions mold the behavior of urban bird species is still a topic open for discussion, but an important body of evidence suggests that bird populations living in highly urbanized areas, where predation, competition, and parasitism can be relaxed, birds tend to show higher boldness. Our results add to that body of evidence. However, future research is needed to untangle the mechanisms behind the strong correlation we found between House Sparrow FIDs and human settlement size.

## Data Availability

The datasets and/or analyses generated during the current study are available from the corresponding authors upon reasonable request.
